# Degree of Skin Denervation and Its Correlation to Objective Thermal Sensory Test in Leprosy Patients

**DOI:** 10.1371/journal.pntd.0001975

**Published:** 2012-12-13

**Authors:** Ismael Alves Rodrigues Júnior, Isabel Cristina Costa Silva, Letícia Trivellato Gresta, Sandra Lyon, Manoel de Figueiredo Villarroel, Rosa Maria Esteves Arantes

**Affiliations:** 1 Departamento de Patologia, Instituto de Ciências Biológicas, Universidade Federal de Minas Gerais, Belo Horizonte, Brazil; 2 Departamento de Dermatologia, Hospital Eduardo de Menezes, Fundação Hospitalar do Estado de Minas Gerais, Belo Horizonte, Brazil; 3 Núcleo de Estudos e Pesquisa em Engenharia Biomédica da Universidade Federal de Minas Gerais, Belo Horizonte, Brazil.; University of California San Diego School of Medicine, United States of America

## Abstract

**Background:**

Leprosy is an infectious disease affecting skin and peripheral nerves resulting in increased morbidity and physical deformities. Early diagnosis provides opportune treatment and reduces its complications, relying fundamentally on the demonstration of impaired sensation in suggestive cutaneous lesions. The loss of tactile sensitivity in the lesions is preceded by the loss of thermal sensitivity, stressing the importance of the thermal test in the suspicious lesions approach. The gold-standard method for the assessment of thermal sensitivity is the quantitative sensory test (QST). Morphological study may be an alternative approach to access the thin nerve fibers responsible for thermal sensitivity transduction. The few studies reported in leprosy patients pointed out a rarefaction of thin dermo-epidermal fibers in lesions, but used semi-quantitative evaluation methods.

**Methodology/Principal Findings:**

This work aimed to study the correlation between the degree of thermal sensitivity impairment measured by QST and the degree of denervation in leprosy skin lesions, evaluated by immunohistochemistry anti-PGP 9.5 and morphometry. Twenty-two patients were included. There were significant differences in skin thermal thresholds among lesions and contralateral skin (cold, warm, cold induced pain and heat induced pain). The mean reduction in the density of intraepidermal and subepidermal fibers in lesions was 79.5% (SD = 19.6) and 80.8% (SD = 24.9), respectively.

**Conclusions/Significance:**

We observed a good correlation between intraepidermal and subepidermal fibers deficit, but no correlation between these variables and those accounting for the degree of impairment in thermal thresholds, since the thin fibers rarefaction was homogeneously intense in all patients, regardless of the degree of sensory deficit. We believe that the homogeneously intense denervation in leprosy lesions should be objective of further investigations focused on its diagnostic applicability, particularly in selected cases with only discrete sensory impairment, patients unable to perform the sensory test and especially those with nonspecific histopathological finds.

## Author Summary

Our study has addressed objectively the rarefaction of cutaneous thin nerve fibers density and its correlation with quantitative thermal sensory test in leprosy patients. Thermal sensitivity evaluation is crucial to the early diagnosis of leprosy, since it is the first type of cutaneous sensitivity lost in the lesions. However, some patients are unable to perform thermal tests, like children and patients with cognitive impairment. The pathological study of those lesions is also fundamental and it is the gold standard method to diagnose and classify leprosy patients. However, it may also be unspecific, especially in the indeterminate form of leprosy. Our findings show that even in lesions with slight thermal sensitivity impairment, there is a homogeneously intense denervation, specifically in the superficial skin layers. We believe that our findings pave the way to future studies focused on the diagnostic applicability of the cutaneous thin nerve fibers density quantification in leprosy suspected lesions.

## Introduction

Leprosy is an infectious disease affecting skin and peripheral nerves [1], [2], [3], [4]. The neural impairment results in increased morbidity and, sometimes, disabling permanent physical deformities. Prompt diagnosis during the incipient stages is important to avoid these complications. The World Health Organization and the Brazilian Ministry of Health guidelines propose leprosy diagnosis based on the detection of skin lesions with impaired sensation, thickened peripheral nerves or a positive skin smear [5], [6], [7].

In the initial phase, the presence of hypochromic macule occurs without neural thickening and with a negative dermal smear, making the sensitivity test of a suspicious lesion an important criterion to establish the early diagnosis. The tactile sensitivity test with Semmes-Weinstein monofilaments is the most common and applicable test among the available sensitivity tests for outpatient setting [8].

In leprosy lesions, loss of tactile sensitivity is preceded by loss of thermal sensitivity, since tactile sensitivity, mediated by thick myelinated A-beta type nerve fibers, can be preserved, even if the loss of thermal sensitivity, mediated by thin myelinated A-delta type fibers and thin unmyelinated C type fibers, has already occurred [9], [10]. Thus, the assessment of thermal sensitivity is of fundamental importance to establish an early diagnosis.

The use of test tubes with hot and cold water is hampered in ambulatory practice since it is time consuming and the water temperature control may be difficult to standardize [11]. The quantitative sensory test (QST) performed by electronic equipment is considered the gold standard method to assess thermal sensitivity [12], [13], [14], [15], [16], despite its large size, high cost and need for an experienced professional.

Morphological study of skin biopsy is an alternative to assess thin nerve fibers structure and densities related to the thermal sensitivity function [17]. The protein gene product (PGP 9.5) is a neuronal pan-axonal marker widely used for intraepidermal and dermal nerve endings analysis and quantification. Current guidelines recommend skin biopsy rather than peripheral nerve biopsy in the diagnosis of thin fiber neuropathies [18] since the methods to evaluate peripheral nerve conduction, which assess thick nerve fibers, may fail to detect nerve impairment [19].

In leprosy patients a few morphological studies showed a decrease of thin cutaneous nerve fibers density associated to the worsening of heat and cold detection thresholds. However, such studies have used non-systematic semi-quantitative methods to evaluate the nerve density and the thermal thresholds, and did not exclude treated patients [20], [21], [22], [23], [24].

## Methods

### Objectives

This work aimed to study the correlation between the degree of thermal sensitivity impairment, measured by QST, and the degree of denervation in leprosy skin lesions, evaluated by immunohistochemistry anti-PGP 9.5 and morphometry.

### Leprosy diagnosis

According to the Brazilian guidelines (27), written in the form of ordinances, a case of leprosy is defined if a patient fulfills one of the criteria: skin lesion with decreased sensitivity, positive skin smear or enlarged peripheral nerve. Although the diagnosis can be based on only one of these criteria, only patients who had skin lesions could be included in our work. In our reference center, at the Eduardo de Menezes Hospital of the Minas Gerais State Hospital Foundation, the tactile sensitivity test of suspicious lesions is performed with dry cotton wool and, for research purposes, monofilaments. The thermal sensitivity test is performed with hot and cold water tubes and, for research purposes, sensory thermo-analyzer. The histamine test is performed in suspicious lesions that showed no significant sensitivity impairment. Skin smears are routinely performed on all suspected or confirmed cases. The pathological examination of the lesions is routinely performed for diagnostic investigation in suspected cases and research purposes in previously confirmed cases. Clinical examination of peripheral nerves is done routinely in suspected and confirmed cases.

### Participants

Leprosy patients with at least one skin lesion with a minimum diameter of three centimeters to offer an adequate docking area for the thermal stimulator were included if they had been in treatment for maximum of 30 days. Patients were excluded if lesions were located in body regions impaired by leprosy peripheral neuropathy, clinically evaluated by peripheral nerves palpation, tactile sensitivity test of the palms and soles, evaluation of muscular trophy and palms and soles hydration. We also excluded patients with other diseases known to cause peripheral neuropathy, such as alcoholism, diabetes, HIV infection, thyroidopathy, metabolic disturbances or systemic vasculitis as well as patients with limited cognitive ability, unable to respond adequately to QST, or whose scars from previous biopsies compromised the skin areas to be studied.

### Description of procedures or investigations undertaken

#### Tactile test and thermal quantitative sensory test

The tactile sensitivity of the lesions was assessed by Semmes-Weinstein monofilaments, a set of nylon rods, with gradually thicker gauges, that pressed on the skin, exert different pressures. The force exerted by the monofilament: green = 0,05 g; blue = 0,2 g; lilac = 2,0 g; dark red = 4,0 g; orange = 10,0 g.

The QST was performed in a quiet environment using the Thermal Sensory Analyzer TSA-II (Medoc, Ramat Yishai, Israel) programmed with a 32°C basal temperature stimulator, a linear variation of 1°C per second, a maximum temperature of 50°C for heat and 0°C for cold, a return to baseline at 0.8°C per second, a programmed interstimulus interval of four to 6 seconds and a thermal stimulator area of 3.0×3.0 cm^2^.

Lesions were tested immediately after contralateral skin for each of the four thresholds determined. The cold (CPT) and warm (WPT) perception thresholds were determined by the method of levels. Then, the cold-induced (CPPT) and heat-induced (HPPT) pain perception thresholds were determined by the method of limits.

An automatic safety restriction of the equipment limited the temperature variation to which patients were exposed to the range of 0°C to 50°C. When the patient did not perceive cold at 0°C or heat at 50°C, these borderline values were recorded, allowing for statistical analysis.

#### Skin biopsy

The comparative thin nerve fibers density in the corresponding contralateral free of lesions skin was essential to study the fibers density in leprosy lesions. The lesions denervation was calculated in relation to the contralateral skin innervation. After determining the thermal thresholds of the affected and contralateral skin, all patients underwent biopsy of the two sites by means of a five-millimeter diameter punch.

#### Processing of skin fragments

The samples were fixed in 10% formalin solution, paraffin-blocked and 10-µm thick sequential sections were obtained. After deparaffined and rehydrated the sections stained with hematoxylin and eosin (HE) and WADE were classified according to Ridley and Jopling leprosy classification [25]. For immunohistochemical procedures endogenous peroxidase activity and nonspecific binding were blocked, followed by incubation in goat serum diluted at 1∶20 in 0.01 M PBS with 0.2% serum albumin and 0.001% Triton X. After blocking, the sections were incubated overnight with anti-PGP 9.5 rabbit polyclonal antibodies (Ultraclone Ltd, England) at a concentration of 1∶400 in antibody diluting solution (Dako, USA) in a moist chamber at 4°C. The slides were washed in PBS and sequentially treated with pre-diluted biotinylated anti-rabbit and anti-mouse immunoglobulin (Dako LSAB® Kit, USA) for 30 minutes and with pre-diluted streptavidin-peroxidase conjugate (Dako LSAB® Kit, USA) for an additional 30 minutes interval. The reaction was revealed in a solution of diaminobenzidine (DAB, Sigma, USA) and 30 volumes hydrogen peroxide in PBS. The sections counterstained with Harris hematoxylin were coverslips-mounted with Entelan (Merck, Germany).

#### Morphometry

Two measurements were determined by morphometry: (1) the count of intraepidermal nerve fibers per millimeter of epidermis; and (2) the ratio between the area of subepidermal nerve fibers marked and the total area of the papillary dermis examined. The images were obtained using an Olympus BX51 optical microscope (Olympus, Japan) and captured by the video color camera Cool SNAP-Probcf (Media Cybernetics, USA) coupled to a computer using the Image-Pro Express 4.0 software (Media Cybernetics, United States). KS300 program (Carl Zeiss Micro Imaging, Germany) was used for analysis of the scanned images.

The intraepidermal nerve fibers were counted by a full scan of both sections of each slide using an objective magnification of 40×. We considered only fibers above the basal cell layer to minimize the erroneous counting of unwanted structures. Panoramic images of each slice, obtained with a 4× objective, were captured to measure the length of the epidermis. Using the KS 300 program, a line was drawn to delineate the full extent of the lower limit of the basal layer providing the epidermis length automatically.

To count the subepidermal fibers stained brown by immunohistochemistry, screening of all papillary dermis in both sections of the slides was performed using an objective magnification of 20×. The marking of unwanted structures such as cutaneous annexes and inflammatory infiltrate prevented the automated counting of nerve fibers in deeper regions of the papillary and reticular dermis in lesions, limiting the nerve fibers quantification to a depth limit of 50 µm from the extremity of the most superficial dermal papillae, both in lesions and contralateral skin. So, in each image, a blueprint was designed to exclude the epidermis and the dermis located deeper than this boundary line. Through the image analysis program, the area corresponding to the structures stained in brown and the total dermal area delineated in the blueprint were automatically obtained in µm^2^ for each field scanned.

### Ethics

The study was approved by the Research Ethics Committee of the Federal University of Minas Gerais and all participants gave their written informed consent.

### Statistical methods

The Statistical Package for Social Sciences (SPSS) 15.0 (SPSS, USA) was used in the descriptive analysis measures of central tendency (mean and median), variability (the standard deviation and coefficient of variation) and percentages.

For comparative analysis between the paired groups “lesions” and “contralateral skin”, nonparametric continuous variables were studied with Wilcoxon test, Kruskal-Wallis test and Spearman correlation coefficient.

Taking as reference the work of Cohen [26], the sample size of twenty-two patients was sufficient to study the correlation aiming a power of 0.8 and accepting as parameters a significance of 0.1 and an effect size of 0.5.

## Results

Between January and December 2009 twenty two new leprosy cases were included in the study. All patients had lesions with thermal hypoesthesia and/or tactile deficit. At least 19 patients showed typical histology of the disease. Three patients had only mild perineural, perivascular and periannexal lymphocytic infiltrate. These three had hypochromic macules with indubitable thermal and/or tactile hypoesthesia. All patients showed improvement of skin lesions after treatment initiation.

Eleven patients were men. The mean age was 42 years (ranging between 10 and 73 years). Ten patients (45.5%) presented with six or more lesions and four (18.2%) had more than one clinically impaired peripheral nerve. According to the WHO operational classification ten patients (45.5%) were considered multibacillary. Under the Ridley-Jopling classification four patients (18.2%) were considered to have the indeterminate form of leprosy, two (9.1%) had the tuberculoid form, five (22.7%) had the borderline tuberculoid form, nine (40.9%) had the borderline lepromatous form and two (9.1%) had the lepromatous form.

**Figure 1:**
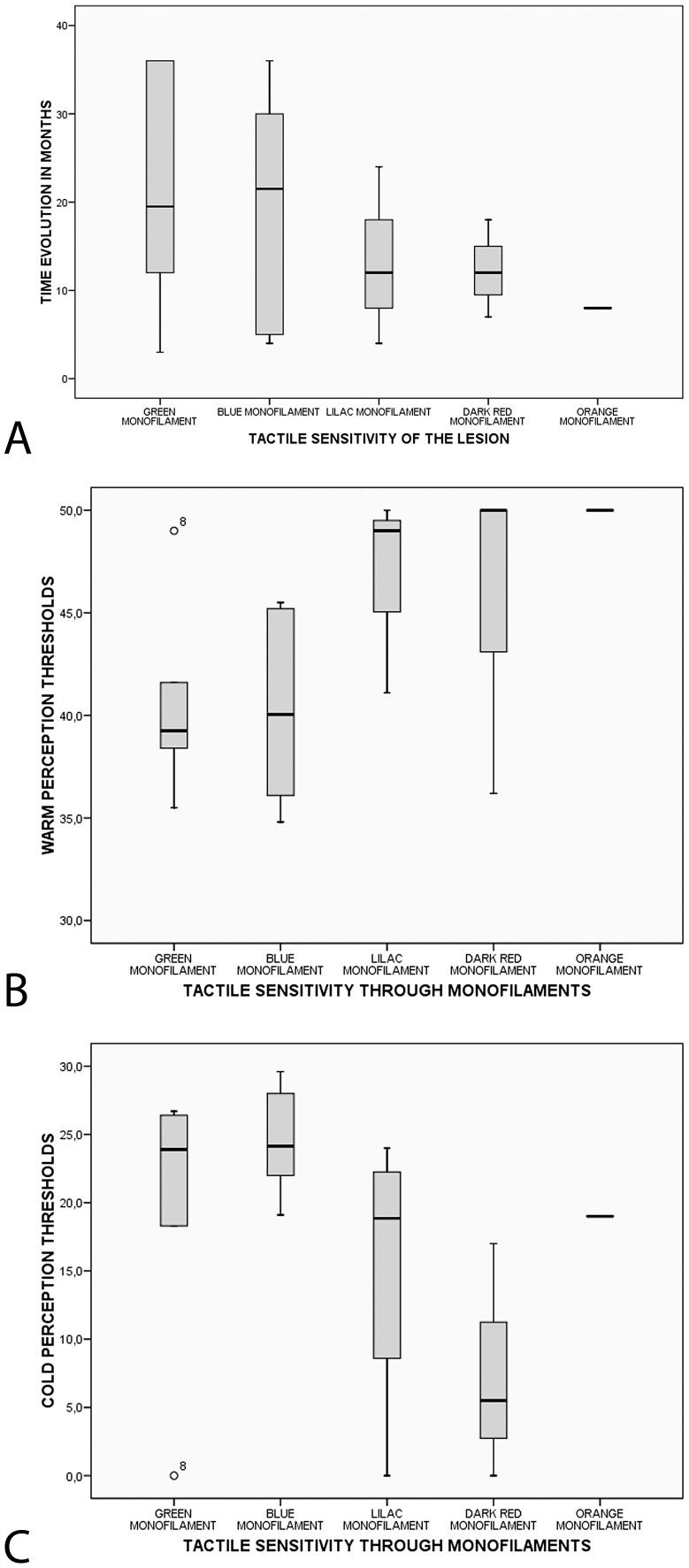
Sensitivity in leprosy lesions. (A) Patients with preserved or just slightly impaired tactile sensitivity in their lesions tended to present a longer evolution time until diagnosis. *p* = 0.759 (Kruskal-Wallis test). (B) Distribution of warm and (C) cold perception thresholds in lesions stratified according to the results of the tactile sensitivity test. There was a trend towards an association between worsening WPT (A) and CPT (B) and the degree of tactile sensitivity loss.

Tactile sensitivity was preserved to the 0.05 g, 0.2 g, 2.0 g, 4.0 g and 10.0 g monofilament in 30%, 30%, 20%, 15% and 5% of the lesions, respectively. The lesions with preserved or mostly preserved tactile sensitivity tended to present a longer evolution time until diagnosis than lesions with a more pronounced loss of tactile sensitivity (Fig. 1A).

QST results were different in “lesions” and “contralateral skin” groups for each of the thermal thresholds evaluated: CPT, WPT, CPPT and HPPT (Table 1). We noticed a trend towards an association between worsening WPT (Fig. 1B) and CPT (Fig. 1C) and the degree of tactile sensitivity loss.

**Table 1:** Quantitative thermal test thresholds and nerve fibers quantification in lesions and contralateral skin.

	Lesion		Contralateral skin			Absolute Difference		Percentage Difference		
Perception threshold	Mean (°C)	Standard deviation	Mean (°C)	Standard deviation	*p*	Mean (°C)	Standard deviation	Mean (%)	Standard deviation	CV*
Cold	19.0	9.22	27.9	2.3	<0.001	9.2	8.5	33.6	32.1	0.95
Warm	43.2	5.6	37.3	4.7	<0.001	6.0	4.3	16.6	12.6	0.76
Cold induced pain	3.0	6.0	8.7	9.2	0.003	5.5	6.7	39.6	46.5	1.17
Heat induced pain	48.9	2.1	44.6	4.2	<0.001	4.2	3.7	10.1	9.4	0.93

Cutaneous fibers	Mean	Standard deviation	Mean	Standard deviation	*p*	Mean	Standard deviation	Mean	Standard deviation	CV*
Intraepidermal (/mm)	1.2	2.3	4.7	3.5	<0.001	3,6	2,9	80,8	24,9	0,30
Subepidermal (%dermis)	0.2	0.2	1.1	0.8	<0.001	0,8	0,7	79,5	19,6	0,24

Notes: *p* value of the Wilcoxon test.

* CV: coefficient of variation (standard deviation/mean).

The loss of thermal sensitivity, interpreted as the absolute and the percentage difference between lesions and contralateral skin is presented in Table 1 and is stratified according to the tactile sensitivity of the lesions in Table 2.

**Table 2:** Stratification of tactile sensitivity and the degree of thermal sensitivity impairment in lesions.

Tactile sensitivity in lesions	Difference in CPT (°C) (contralateral skin - lesion)			Difference in WPT (°C) (lesion - contralateral skin)		
	Mean	Minimum	Maximum	Mean	Minimum	Maximum
Green	8.1	2.3	27.6	4.3	1.0	7.5
Blue	3.8	1.0	6.2	5.1	1.3	12.4
Lilac	12.1	3.8	25.9	9.0	4.6	13.0
Dark red	18.8	7.0	25.0	7.7	3.2	15.4
Orange	8.2	-	-	5.4	-	-

Note: The thermal thresholds impairments were calculated as the percentage difference between the values obtained in the skin and the contralateral lesion. The degree of thermal sensitivity impairment in lesions was compared to contralateral skin and indicated by the absolute difference between the threshold values.

**Figure 2:**
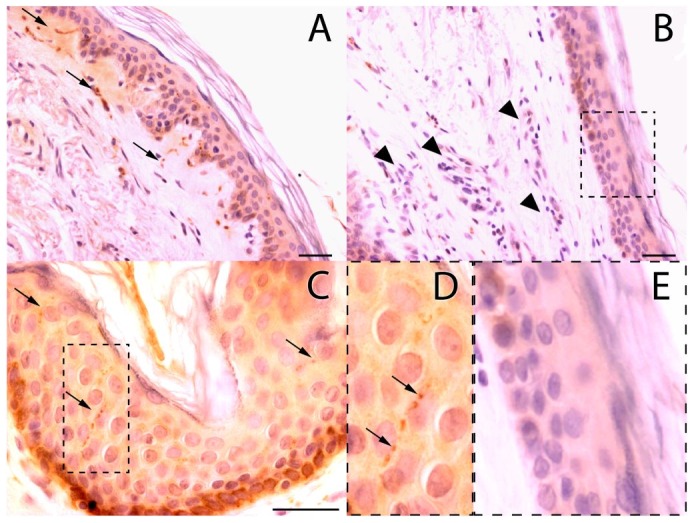
Immunohistochemical staining for PGP 9.5 in leprosy patients. (A) Subepidermal fibers in the contralateral skin (arrows). (B) Inflammatory infiltrate (arrowheads) in the lesion skin. Notice the scarcity of stained nerve fibers. (C) Intraepidermal fibers (arrows) in the contralateral skin are demarked with a dotted line in (D) showing visible nerve endings varicosities (arrows). (E) Notice the scarcity of intraepidermal fibers in the magnified field of the lesion skin. Bar = 30 µM.

The subepidermal nerve fibers in contralateral biopsies were visualized as thicker brown structures with variable diameter and density of staining. These fibers were linear or sometimes grouped in small clumps, placed parallel to the basal layer and sometimes positioned to cross it towards the surface (Fig. 2A). The contralateral skin biopsies also showed intraepidermal nerve fibers as discontinuous linear structures stained in brown, resembling “beads” that vertically crossed the basal, spinous and granular layers toward the skin surface, then bent and continued parallel to the corneum stratum, inside of which they could eventually be seen (Fig. 2C and D). However, not all intraepidermal fibers were visualized throughout their entire course. Often, the fibers crossed the section obliquely and were only seen in portions of its course.

Lesion biopsies showed a substantial decrease in subepidermal fibers (Fig. 2B, arrowheads) and intraepidermal fibers (Fig. 2E).

Evaluation of intraepidermal and subepidermal denervation was performed in each patient by determining the percentage difference between the values obtained in the lesion and the contralateral skin. The lesions presented a mean rarefaction of subepidermal fibers of 79.5% (SD = 19.6; coefficient of variation = 0.24) and a mean reduction of intraepidermal fibers of 80.8% (SD = 24.9; coefficient of variation = 0.30) compared to the contralateral areas (Table 1).

Good correlation was detected between the deficit of intraepidermal fibers and the deficit of subepidermal fibers (Spearman coefficient: 0.60; *p* = 0.004). However, the bivariate analysis showed lack of statistical correlation between intraepidermal or subepidermal fibers deficit and thermal thresholds deficit.

## Discussion

The Ministry of Health of Brazil recommends the use of a thin dry cotton swab to detect the tactile sensitivity deficit in leprosy lesions [27]. For the evaluation of protective sensation in hands and feet, some authors consider the inability of perception of the 0.2 g [28] or 2.0 g [29] monofilaments as the definition of tactile impairment. However, there is still no consensus regarding the threshold to be considered when the monofilaments are used in the assessment of tactile sensation on suspicious skin lesions for leprosy diagnostic purposes.

In our hands the diagnosis was properly performed before the patient was enrolled in the study. We tested lesions with the monofilament test in order to enable quantification of tactile lesions, since the test with cotton swab is only a qualitative test. We wanted to counterbalance the quantitative result (or semi-quantitative) of monofilament to the quantitative results of thermal sensitivity and quantification of morphological innervation of the skin. In fact, the use of monofilaments has been previously studied by our group and compared to the quantitative thermal test for the examination of suspicious lesions of Leprosy [30].

In 30% of our patients, the lesions were sensitive to the 0.05 g monofilament and in 30% the tactile sensitivity deficit was mild, with preserved sensitivity to the 0.2 g monofilament. The diagnosis of leprosy may be challenging when lesions do not present tactile impairment or when only a slight tactile hypoesthesia is demonstrated.

That may explain the longer period of evolution before diagnosis in patients presenting with normal or only small changes in tactile sensitivity. Outside the referral service, the evaluation of suspected lesions seems so strongly dependent on tactile tests that late diagnosis may occur if the Semmes-Weinstein test is normal or slightly impaired.

The thermal sensitivity deficit in leprosy lesions is known to precede the loss of tactile sensitivity [28], [31]. In our hands, all lesions with a preserved tactile sensitivity to the 0.05 g or 0.2 g monofilaments showed impaired CPT and WPT. The most significant difference between lesions and contralateral skin was in the CPT, with mean absolute difference of 9.2°C. Averagely, the difference for the WPT was 6.0°C. The more significant impairment in CPT compared to WPT in leprosy lesions has been demonstrated by other studies, suggesting a more intense involvement of thin myelinated type A-delta fibers than thin unmyelinated type C fibers in leprosy skin lesions [30].

The quantification of contralateral skin intraepidermal fibers showed a mean of 4.7 fibers per millimeter of epidermis considering all sites examined. This value is similar to the mean of 5.3 found in a Brazilian study focused on the standardization of intraepidermal fibers quantification in the distal leg of healthy volunteers [32]. Nevertheless, it is lower than those found by most other authors. For instance, the quantification of intraepidermal fibers from the healthy distal leg showed means of 9.6/mm [33], 16.3/mm [34], 11.1/mm [35], 17.8/mm [36], 13.8/mm [37] and 5.2/mm [38]. For the proximal region of the thigh, normal means of 21.1/mm [37], 20.3/mm [33] and 23.8/mm [34] have been reported. To date, we do not have knowledge of reports on the standardization of intraepidermal fiber density in other regions besides those on lower limbs, used in the study of thin fiber neuropathies. Thus, ours and other previously published data point out for the need of standardization of normal fiber density in different body localization.

Intraepidermal fibers were seen in contralateral free of lesions skin of all patients, contrasting authors [23] who reported a complete intraepidermal denervation in 16 of 28 biopsies of contralateral skin in leprosy patients. This may be due to the counting criteria adopted. In our study, shorter segments, representing fibers arranged obliquely to the cutting plane of the paraffin block, were counted. Still, the contralateral skin of our patients showed a reduction of intraepidermal fibers when the reference values mentioned previously [33], [34], [35], [36], [37], [38] were taken into account.

This intraepidermic denervation may be an evidence of systemic pathogenic mechanisms acting beyond the topographically limited inflammatory infiltrate typical of leprosy. Antunes and others have observed that no exact correspondence exists between the denervation regions and those affected by inflammatory infiltrate in lesions [21]. Facer and others have proposed that the thinning in intraepidermal fibers in the contralateral skin could be related to a widespread reduction in nerve growth factor [22].

In our study, intraepidermal fibers were absent in the lesions of six patients. In other sixteen, they were significantly reduced, but not completely abolished. This finding differs from others who have observed a complete intraepidermic denervation in lesions of all [23] or almost all patients [39]. Again, this may be due to the standardization criteria adopted for fiber counting. We believe that even short segments of intraepidermal fibers must be quantified because these segments are representative of fibers arranged obliquely to the cutting plane of the material.

To calculate the deficit in thermal thresholds and cutaneous innervation, we chose to use the percentage difference between contralateral skin and lesions. This should counteracts the physiological variations depending on the body region studied [40], [41], [42], [43]. The threshold temperatures vary regionally, depending on factors such as thickness of epidermis, physiological fluid volume and density of cutaneous innervation [44]. Likewise, the density of intraepidermal innervation presents important regional variation, with respect to the craniocaudal concentration gradient, higher in the trunk than in the limbs and higher in their proximal region than at their distal extremities [45].

The denervation observed in lesions was considerable. On average, the lesions showed 20.5% of the subepidermal fibers and 19.2% of the intraepidermal fibers found in the contralateral skin biopsies. This marked denervation was homogeneous among all patients, especially regarding the subepidermal fibers. The coefficients of variation of the percentage differences in subepidermal and intraepidermal fibers were 0.24 and 0.30, respectively, indicating homogeneity in their deficit among the patients. In turn, the coefficients of variation of the percentage differences in WPT, CPT, CPPT and HPPT were high (0.95, 0.76, 1.17 and 0.93, respectively), indicating important variation in their deficit in our sample.

Although seemingly obvious, a correlation could not be detected between cutaneous denervation and thermal sensitivity impairment. The observation of those coefficients of variation anticipated that the degree of sensory deficit had been significantly variable among the patients, whereas the decrease in thin cutaneous fibers had been quite uniform in all cases.

Otherwise, the same significant and homogeneous rarefaction of innervation may support the use of quantification of thin cutaneous neural fibers as an additional diagnostic tool for leprosy. Thermal sensitivity tests are known to be dependent upon the patient's ability to respond correctly to the instructions of the examiner, which may hinder its application in children or in patients with cognitive impairment. The histological study of a suspicious lesion, considered the gold standard for establishing the diagnosis, may also be nonspecific [46]. These difficulties have not been fully addressed in other tests performed on lesion biopsies. The search for the causative microorganism using *in situ* hybridization or polymerase chain reaction, for example, have only demonstrated sensitivities of 45.5% and 66.6%, respectively [47]. In this regard, the objective quantification of thin fibers in the skin of patients with suspicious skin lesions should be the object of larger studies specifically aimed at its validation within the existing methods for leprosy diagnosis.

### Limitations

The lack of correlation between cutaneous denervation and thermal sensitivity impairment may arise from the fact that variations in thermal thresholds are also related to changes in the innervation of deeper skin layers that we did not approach in the current study. However, it should be considered that we have not yet conducted the qualitative ultrastructural analysis of nerve endings, the investigation of the immunocytochemical expression of the several neurotransmitters involved in thermal sensitivity control, or the study of inflammatory cytokines expression, factors that may be related to the functional impairment of cutaneous nerve endings [48], [49].
